# Association between plasma homocysteine levels and cognitive deficits in Han Chinese patients with schizophrenia across age groups

**DOI:** 10.1038/s41598-021-99239-3

**Published:** 2021-10-05

**Authors:** Sumiao Zhou, Yuanyuan Huang, Yangdong Feng, Hehua Li, Kai Wu, Mingzhe Yang, Fengchun Wu, Xingbing Huang

**Affiliations:** 1 Department of Psychiatry, The Affiliated Brain Hospital of Guangzhou Medical University, Liwan District, Guangzhou, 510370 China; 2 Department of Biomedical Engineering, School of Materials Science and Engineering, South China University of Technology (SCUT), Guangzhou, China; 3 Guangdong Engineering Technology Research Center for Translational Medicine of Mental Disorders, Guangzhou, China

**Keywords:** Human behaviour, Psychology, Diseases

## Abstract

It was still unclear how homocysteine (Hcy) levels and cognitive deficits change in patients with schizophrenia of various ages. The present article attempts to assess the relationship between Hcy levels and cognitive deficits in patients with schizophrenia across age groups, especially in young people. Totals of 103 patients and 122 healthy controls were included. All participants were stratified into four groups according to their age: 18–29 years, 30–39 years, 40–49 years, and 50–59 years. Clinical data, plasma Hcy levels, and cognitive function score were collected. Cognitive function was evaluated using the MATRICS Consensus Cognitive Battery of tests assessing speed of processing, verbal learning and memory, visual learning and memory, working memory, and attention/vigilance. Compared with the healthy group, Hcy levels increased significantly, and all the measured cognitive function score were significantly lower in all age groups of patients with schizophrenia (*p* < 0.001). Hcy levels were negatively associated with speed of processing (SoP), working memory (WM), and visual learning and memory (Vis Lrng) score in 18–29 years. Further multiple regression analysis showed that SoP were independently associated with Hcy levels in patients with schizophrenia aged 18–29 years (B = 0.74, t = 3.12, p = 0.008). Based on our results, patients with schizophrenia performed worse on cognitive assessments and Hcy levels were more closely related to cognition in young patients.

## Introduction

Schizophrenia is a severe psychiatric syndrome characterized by psychotic symptoms, negative symptoms and cognitive deficits. It is among the top ten global causes of disability^1^. Biomarkers, such as homocysteine (Hcy), kynurenic acid, C-reactive protein, and triiodothyronine, have been shown to be potential risk factors for schizophrenia^2–5^. Hcy interacts with NMDA receptors, induces apoptosis, triggers oxidative stress, injures mitochondrial function and leads to vascular damage, which played an essential role in the occurrence and development of schizophrenia^6^. Elevated plasma Hcy levels might also contribute to cognitive deficits that are common in patients with schizophrenia, even when the analyses are corrected for the relevant covariates^7^. However, several epidemiological studies have shown that elevated Hcy levels do not appear to be correlated with cognitive deficits in patients with schizophrenia^8^. Therefore, the association between Hcy levels and cognitive deficits in patients with schizophrenia continues to be debated.

Abnormal accumulation of Hcy in plasma is a high-risk factor for neurodegeneration in individuals aged 65 years^9^. Moreover, a previous study showed that plasma Hcy levels increased significantly with age in a Northeast Chinese population^10^. In addition, Chen et al.^6^ showed a significant association between higher plasma Hcy concentration and worse cognitive performance in patients with late-onset bipolar disorder. Taken together, age may play an important role in determining Hcy levels in both the healthy population and patients with psychiatric disorders. However, we do not know the association between Hcy levels and cognitive deficits in patients with schizophrenia across of various ages.

Based on the increased plasma Hcy levels and cognitive deficits in patients with schizophrenia, as well as the close relationship between Hcy levels and age, we were very interesting in exploring their possible association in patients with schizophrenia. To the best of our knowledge, no study has reported the association between Hcy levels and cognitive deficits in patients with schizophrenia across age groups. Therefore, there are two purposes for the present study: (1) to determine whether differences in plasma Hcy levels exist in patients with schizophrenia across age groups by performing a statistical analysis and (2) to determine whether plasma Hcy levels and cognitive deficits are correlated in patients with schizophrenia from different age groups.

## Methods

### Subjects and procedures

Totals of 103 patients who met DSM-V diagnosis for schizophrenia were recruited from the inpatient department of the Affiliated Brain Hospital of Guangzhou Medical University. All patients were conducted from December 2018 to May 2019. The patients met the following criteria: (a) Han Chinese, age 18–59 years, (b) with a duration of illness of at least 5 years and at least 5 years of education, (c) a diagnosis conducted by an independent experienced psychiatrist on the basis of the Structured Clinical Interview for DSM-IV (SCID), and (d) take a stable dose of antipsychotic drugs for ≥ 4 weeks before the start of the study.

The exclusion criteria were as follows: (a) use of immunosuppressive agents; (b) past history of autoimmune disorders; (c) substance dependence/abuse; (d) breastfeeding or pregnancy; and (e) major medical abnormalities, including central nervous system diseases and cancer.

Totals of 122 healthy individuals were recruited from community. All subjects were recruited during the same period from Guangzhou. None of them had serious physical diseases or drug abuse/dependence, excluding nicotine.

### Psychotic symptoms

The Positive and Negative Syndrome Scales (PANSS) were performed to assess the clinical symptoms of all patients by two psychiatrists who participated in a training course. Repeated assessment found that the interrater correlation coefficient remained ≥ 0.80 for the PANSS total score.

### Cognitive assessment

Cognitive function was evaluated using the MATRICS Consensus Cognitive Battery (MCCB) of tests assessing speed of processing (SoP), working memory (WM), verbal learning and memory (Vrbl Lrng), visual learning and memory (Vis Lmg), and attention/vigilance (AV). The two researchers participated in a training course in the use of MCCB to ensure the consistency and reliability of ratings. They maintained an interrater correlation coefficient of 0.85 on the MCCB in repeated assessments.

### Hcy analysis

Plasma samples were obtained to analyze plasma Hcy levels on the same day as the cognitive function assessments. Peripheral venous blood was collected from the participants after at least 8 h of fasting. Plasma Hcy levels were measured using the enzyme cycling assay and analyzed by a technician from Affiliated Brain Hospital of Guangzhou Medical University.

### Statistical analysis

Clinical and demographic data were analyzed using analysis of variance (ANOVA) for continuous variables and the chi-square test for categorical variables. If the ANOVA results were significant, we then performed an analysis of covariance (ANCOVA) of cognitive deficits and controlled for the confounding effects of age, BMI, and education. Bonferroni test was used to perform post-hoc pair-wise comparisons between groups. Second, we separately determined associations between plasma Hcy levels and cognitive deficits in stable patients with schizophrenia across age groups. Finally, multiple regression analyses was used to qualitify the correlations between plasma Hcy levels and cognitive deficits in patients with schizophrenia after adjustment for age, BMI, and education. The validity of the final regression model was established using normal probability plots to identify potential outliers and detect any deviation from the model assumptions. PP-plots and histograms of residuals were assessed for normal distribution in final models. Tolerances and Variance Inflation Factors were closely monitored to detect for multicollinearity. SPSS version 22.0 were used to do all data, and statistical significance was defined as *p* ≤ 0.05 (two-sided).

### Compliance with ethical standards

All procedures were performed in accordance with the Declaration of Helsinki issued by the National Institutes of Health. The study was approved by the ethics committee of the Affiliated Brain Hospital of Guangzhou Medical University. Informed consent form was obtained from each participant. It is worth noting that all participants were competent enough to give their informed consent.

## Results

### Differences in demographics, cognitive function and Hcy levels between patients and healthy controls

Totals of 103 patients and 122 healthy controls were analyzed. Table 1 shows significant differences in age, BMI, and education between patients with schizophrenia and healthy controls (*p* < 0.001). Thus, we controlled for age, BMI, and education in subsequent analyses.

**Table 1:** Sociodemographic data and clinical profiles of patients with schizophrenia and healthy controls.

Variables	Schizophrenia n = 103	Healthy controls n = 122	F/X^2^	*p*
Male	56 (54.4%)	58 (47.9%)	0.92	0.337
Age (years)	42.89 (11.83)	35.02 (12.87)	12.87	< 0.001
BMI (kg/m^2^)	24.56 (4.35)	22.44 (3.44)	3.44	< 0.001
Education (years)	10.89 (3.21)	14.83 (3.05)	3.05	< 0.001
Hcy (mmol/L)	16.28 (10.66)	9.53 (4.63)	4.63	< 0.001
SoP score	33.36 (14.80)	49.85 (9.15)	9.15	< 0.001
WM score	37.64 (13.44)	47.97 (9.75)	9.75	< 0.001
Vrbl Lrng score	33.77 (12.34)	44.27 (10.23)	10.23	< 0.001
Vis Lrng score	36.14 (11.88)	48.07 (10.35)	10.35	< 0.001
AV score	37.73 (10.38)	48.67 (10.30)	10.30	< 0.001
P subscore	10.81 (4.71)	–	–	–
N subscore	17.00 (7.68)	–	–	–
G subscore	28.12 (8.19)	–	–	–
PANSS total	55.82 (16.84)	–	–	–

*Hcy* homocysteine, *SoP* speed of processing, *WM* working memory, *Vrbl Lrng* verbal learning and memory, *Vis Lmg* visual learning and memory, *AV* attention/vigilance, *P* positive symptom, *N* negative symptom, *G* general psychopathology syndrome, *PANSS* the Positive and Negative Syndrome Scales.

In the patient group, all individuals were stratified into four groups according to age: ≤ 18–29 years (n = 17), 30–39 years (n = 19), 40–49 years (n = 32), and 50–59 years (n = 35). Significant differences were observed in Hcy (*p* = 0.022), SoP score (*p* = 0.022) and WM score (*p* = 0.016) after we controlled for age, BMI, and education in subsequent analyses in Table 2. Similarly, all individuals were also stratified into four groups according to age in healthy controls: ≤ 18–29 years (n = 53), 30–39 years (n = 23), 40–49 years (n = 21), and 50–59 years (n = 25). There were no significant differences in Hcy, SoP score, WM score, and Vis Lrng score across age groups, while significant differences were observed in AV score (*p* < 0.001) and Vrbl Lrng score (*p* = 0.046) in healthy controls.

**Table 2:** Clinical profiles of schizophrenia patients across ages group.

Variables	Total (n = 103)	18–29 years (n = 17)	30–39 years (n = 19)	40–49 years (n = 32)	50–59 years (n = 35)	F/X^2^	*p*
Hcy (mmol/L)	13.64 (6.03)	11.07 (3.63)	16.68 (12.04)	16.68 (12.04)	20.02 (12.32)	3.36	0.022
SoP score	29.76 (13.06)	36.74 (12.88)	39.94 (12.06)	39.94 (12.06)	27.26 (16.27)	5.50	0.002
WM score	34.35 (15.37)	38.58 (11.33)	43.28 (11.49)	43.28 (11.49)	33.57 (13.77)	3.06	0.016
Vrbl Lrng score	30.76 (13.83)	32.63 (14.56)	37.25 (12.09)	37.25 (12.01)	32.66 (10.27)	1.15	0.332
Vis Lrng score	36.71 (13.25)	36.05 (14.23)	39.84 (11.78)	39.84 (11.79)	32.51 (8.98)	2.16	0.097
AV score	38.29 (8.45)	39.95 (11.44)	37.53 (11.45)	37.53 (11.45)	36.43 (9.81)	0.31	0.816

*Hcy* homocysteine, *SoP* speed of processing, *WM* working memory, *Vrbl Lrng* verbal learning and memory, *Vis Lmg* visual learning and memory, *AV* attention/vigilance.

### Changes in cognitive deficits and Hcy levels in schizophrenia and healthy controls across age groups

**Figure 1:**
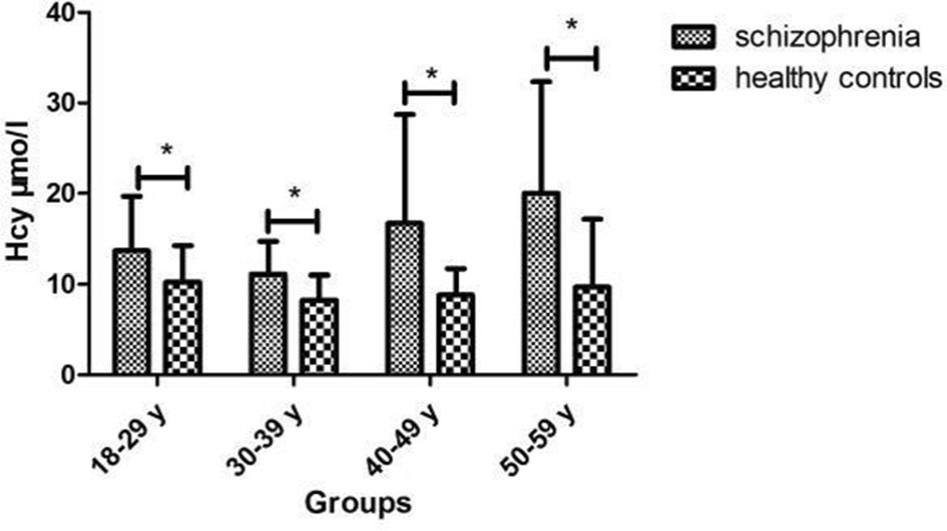
Differences in the plasma Hcy levels between patients with schizophrenia and healthy controls across age groups. *Significant differences in Hcy levels were observed between patients with schizophrenia and healthy controls (*p* < 0.05). *Hcy* homocysteine.

Plasma Hcy levels were significantly lower in patients with schizophrenia than in healthy controls (*p* < 0.001). As shown in Fig. 1, the highest plasma Hcy levels were detected in the 50–59 years group and the lowest levels were detected in the 30–39 years group (*p* = 0.017). Compared with healthy controls, significantly higher Hcy levels were observed in patients with schizophrenia across all age groups, which remained significant after adjustment for age, BMI, and education (all *p* < 0.05).

**Figure 2:**
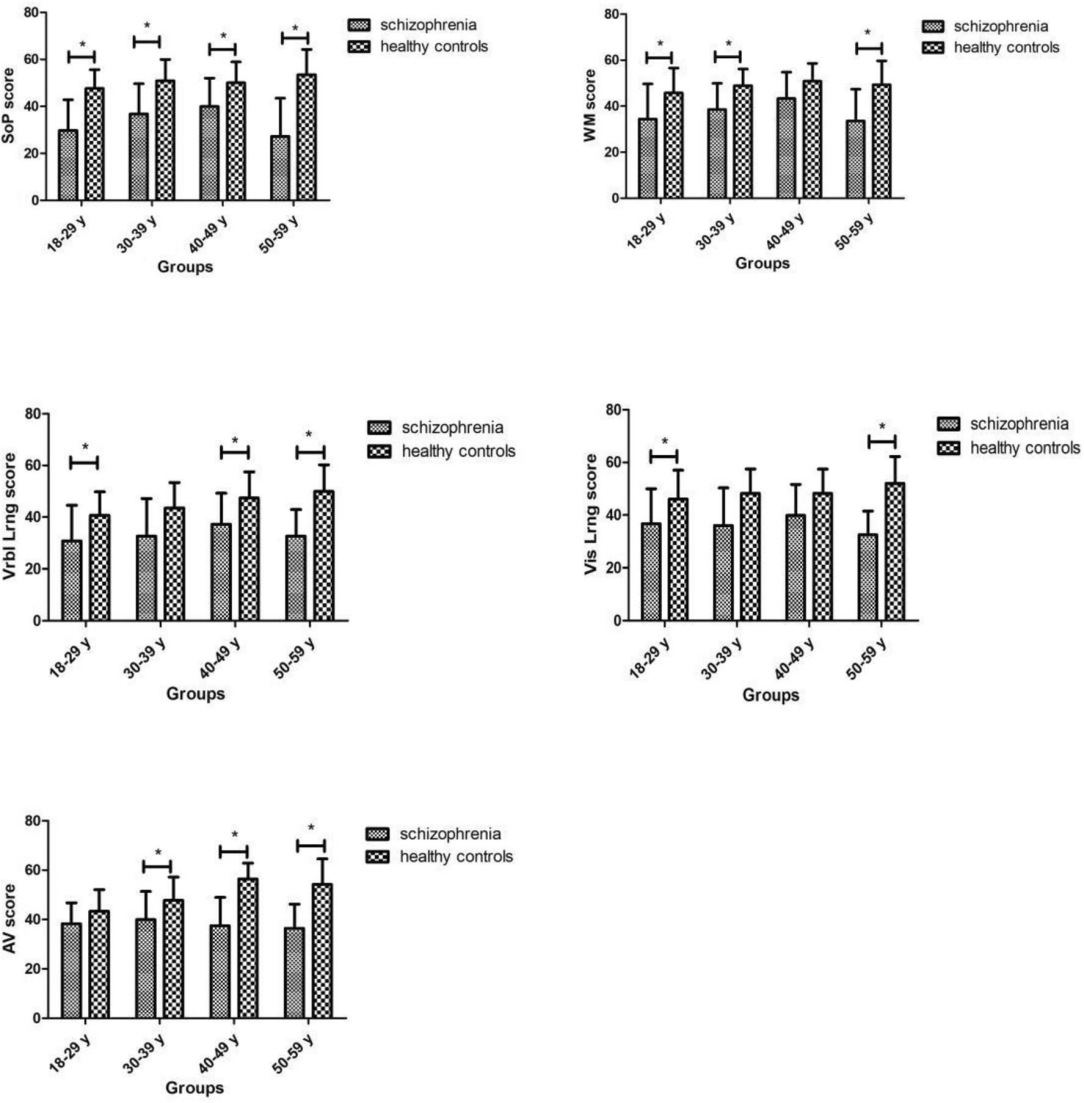
Differences in cognitive test score between patients with schizophrenia and healthy controls across age groups. *Significant differences in cognitive function score were observed between patients with schizophrenia and healthy controls (*p* < 0.05). *SoP* speed of processing, *WM* working memory, *Vrbl Lrng* verbal learning and memory, *Vis Lmg* visual learning and memory, *AV* attention/vigilance.

Figure 2 shows significant differences in cognitive test score for the five indexes of MCCB between patients with schizophrenia and healthy controls among the four groups, which remained significantly after adjustment for age, BMI, and education, except for 40–49 years in WM score (*p* = 0.103), 30–39 years in Vrbl Lrng score (*p* = 0.199), 30–39 years and 40–49 years in Vis Lrng score (*p* = 0.087, *p* = 0.084), and 18–29 years in AV score (*p* = 0.195). The lowest SoP, WM, Vis Lrng, and AV score were recorded by patients aged 50–59 years, and the lowest Vrbl Lrng score were recorded by patients aged 18–29 years.

### Age-related differences in the relationships between Hcy levels and clinical symptoms and cognitive deficits in schizophrenia

Table 3 shows the correlations between Hcy levels, clinical symptoms and cognitive deficits in patients. As shown, plasma Hcy levels showed negatively correlated with SoP, WM, and Vis Lrng score in schizophrenia aged 18–29 years (r = − 0.60, *p* = 0.011; r = − 0.62, *p* = 0.008; and r = − 0.50, *p* = 0.039, respectively). In addition, Hcy levels were negatively correlated with Vrbl Lrng score in patients aged 40–49 years (r = − 0.48, *p* = 0.015).

**Table 3:** The correlations between the plasma Hcy levels and clinical symptoms or cognitive deficits in patients with schizophrenia across age groups.

Group	SoP score	WM score	Vrbl Lrng score	Vis Lrng score	AV score	P subscore	N subscore	G subscore	Total PANSS score
18–29 years									
r	− 0.60	− 0.62	− 0.09	− 0.50	0.44	0.03	0.14	0.14	0.15
*p*	0.011*	0.008*	0.722	0.039*	0.08	0.913	0.592	0.595	0.576
30–39 years									
r	− 0.04	− 0.11	− 0.04	− 0.25	− 0.06	0.22	0.33	0.30	0.40
*p*	0.869	0.662	0.864	0.301	0.819	0.366	0.168	0.216	0.094
40–49 years									
r	− 0.14	0.05	− 0.48	− 0.30	− 0.19	0.33	− 0.01	0.16	0.13
*p*	0.440	0.779	0.005*	0.099	0.299	0.070	0.950	0.375	0.48
50–59 years									
r	− 0.06	0.07	− 0.12	− 0.11	− 0.19	0.01	− 0.17	− 0.17	− 0.17
*p*	0.726	0.694	0.498	0.534	0.276	0.964	0.345	0.327	0.324

*Hcy* homocysteine, *SoP* speed of processing, *Vrbl Lrng* verbal learning and memory, *Vis Lmg* visual learning and memory, *WM* working memory, *AV* attention/vigilance, *P* positive symptom, *N* negative symptom, *G* general psychopathology syndrome, *r* Pearson’s correlation coefficient, *PANSS* the Positive and Negative Syndrome Scales.

*p < 0.05.

After adjusting for confounding factors (including age, BMI, and education), a further multiple regression analysis performed in the subgroup of patients with schizophrenia showed independent associations between SoP and Hcy levels (B = 0.74, t = 3.12, *p* = 0.008) in patients with schizophrenia aged 18–29 years. However, no significant relationships were observed between Hcy levels and Vrbl Lrng score in patients aged 40–49 years.

## Discussion

To our best knowledge, the report is firstly explore the age-specific difference in Hcy levels and the correlation with cognitive deficits in schizophrenia. Our study reported several major findings: (1) higher plasma Hcy levels were detected in patients with schizophrenia than in healthy controls across age groups. (2) Significant differences in MCCB test score were observed for schizophrenia compared to healthy controls among the four age groups. (3) With increasing age, the incidences of increased Hcy levels were not significantly increased. (4) More interestingly, we observed age-related differences in the relationships between increased plasma Hcy levels and cognitive deficits in patients with schizophrenia. Significant relationships between SoP, WM, and Hcy levels were only observed in the 18–29 year age group but not in the other subgroups.

Regland et al. first detected the Hcy levels, and high levels were detected in 45% of patients with schizophrenia^11^. Similar to our results, the Hcy levels were increased in schizophrenia compared to healthy controls, even across age groups. This result is consistent with findings supporting that hypothesis that Hcy levels might be confounding factors in schizophrenia ^12^.

Haidemenos et al. defined schizophrenia as a disease characterized by generalized cognitive deficits^13,14^. Consistent with previous research, our study showed that patients with schizophrenia exhibited significantly worse performance on neurocognitive tests than healthy controls in most age groups^15^, which suggests the occurrence of cognitive deficits in the early course of the disease^16^.

As shown in the present study, the Hcy concentration first decreased and then increased, as the lowest levels were at 30–39 years of age and significantly increased after 40 years of age. In several longitudinal studies, the progression of changes in Hcy levels associated with age was not confirmed. Additionally, a recent large-sample study including 7872 subjects evaluated the age-related differences in Hcy levels in a general Chinese population and showed that the Hcy levels first decreased, then increased, and significantly increased after 50 years of age^17^, similar to the results of our study. However, some previous studies did not observe a statistically significant association between Hcy levels and age^18^, whereas other studies reported that plasma Hcy levels increased significantly with age in the general population and patients with schizophrenia^8,10^. The inconsistent results for this association are unclear. One possible reason may be due to the differences in subjects who had suffered from different diseases, BMIs, regions of residence, races, dietary habits, genetic backgrounds or courses of illness^19–22^; for example, the subjects in the present study were inpatients, and most had suffered from schizophrenia for a long time.

Regarding cohort studies, some recent studies in humans displayed that an increase in the Hcy concentration was negatively associated with the evolution of cognitive deficits in the general population and patients with Alzheimer's disease and schizophrenia, particularly in studies of elderly individuals^7,23,24^. Interestingly, we found that Hcy levels were negatively associated with SoP score, but not score for other cognitive domains, in patients with schizophrenia aged 18–29 years after adjustment for age, BMI, and education. Additionally, with increasing age, no relationship was observed between these factors, in contrast most previous studies^17,25,26^. The inconsistent results may be because plasma Hcy levels result from a complex process mediated by the interaction between genetic and environmental factors, and the independent factors associated with Hcy levels and cognitive deficits differed according to age^27^. Moreover, the negative effects of various risk factors on cognitive deficits increase with aging^16^. Although the mechanism remains unclear, these findings indicated that age-related changes in cognitive deficits in patients with chronic schizophrenia are subtle and not uniform across multiple cognitive domains. These age-related differences must be considered when applying strategies to prevent cognitive deficits.

## Limitations

Several limitations of the present study should be mentioned. One significant drawback of our study is that it was a cross-sectional study and was unable to determine the direct causal relationship between cognitive function and Hcy levels in patients with schizophrenia across age groups. Second, all patients in this study were diagnosed with chronic schizophrenia, limiting the generalizability in other disease states, including first-episode and drug-naive schizophrenia. Third, the demographic data (including age, BMI, educational level, etc.) did not matched between schizophrenia and healthy control groups. Furthermore, the number of patients aged 18–29 years (17) is insufficient to perform a multiple regression. Lastly, Hcy levels and cognitive deficits may be related to many confounding factors, such as dietary patterns, lifestyle, antipsychotic treatment and disease duration. Unfortunately, we did not collect these data in our current study, and further investigation is warranted.

## Conclusions

Based on our results, patients with schizophrenia exhibited cognitive deficits and Hcy levels were more closely related to cognition in young patients than in other subgroups. Therefore, researchers should focus on the complex age–Hcy interaction, and further research is warranted to investigate the mechanisms underlying the effect of Hcy levels on cognitive deficits in different age groups.
